# Nationwide Population-Based Study About Patterns of Prescription Opioid Use and Misuse Among Young Adults in Spain

**DOI:** 10.3389/ijph.2022.1604755

**Published:** 2022-08-19

**Authors:** Pilar Carrasco-Garrido, Carmen Gallardo-Pino, Isabel Jiménez-Trujillo, Valentín Hernández-Barrera, Soledad García-Gómez-Heras, Lidiane Lima Florencio, Domingo Palacios-Ceña

**Affiliations:** ^1^ Department of Medical Specialties and Public Health, Health Sciences Faculty, Universidad Rey Juan Carlos, Alcorcón, Spain; ^2^ Department of Basic Health Sciences, Health Sciences Faculty, Universidad Rey Juan Carlos, Alcorcón, Spain; ^3^ Department of Physical Therapy, Occupational Therapy, Rehabilitation and Physical Medicine, Health Sciences Faculty, Universidad Rey Juan Carlos, Alcorcón, Spain

**Keywords:** public health, young adults, prescription opioids, misuse, drugs survey

## Abstract

**Objective:** Prescription opioid misuse has become one of the most common ways drugs are consumed among young adults. The objective of our study was to describe the prevalence and factors associated with prescription opioid use and misuse among young adults living in Spain.

**Methods:** A nationwide, cross-sectional epidemiological study on the use and misuse of prescription opioids in Spanish Youngers. We used individualized secondary data provided by the Household Survey on Alcohol and Drugs in Spain 2017–2018.

**Results:** Prevalence of prescription opioid use among young adults was 4.89%. Misuse among this population reached prevalence values of 13.4%, with higher values observed among women . The variables associated with a greater probability of prescription opioid use and misuse were misuse of tranquilizers, sedatives, and sleeping pills, along with using cannabis and other illicit psychoactive drugs (aOR = 2.99; 95% CI: 1.10–8.15).

**Conclusion:** Prescription opioid use and misuse in Youngers has important implications for the Spanish public health system, because, even though not currently comparable to the situation in other countries, this drug use could be on the verge of creating similar problems.

## Introduction

During the last decade, interest in studying prescribed medicine misuse has increased, especially after observing the opioid epidemic in the US, where the problem is currently considered a public health emergency [1–3]. According to National Survey on Drug Use and Health (NSDUH) data, in the year 2000 approximately 9.5 million Americans aged 12 or older (3.4% of total population) misused opioids [4]. The NSDUH defines prescription medication misuse as “the use of any drug in a manner other than how it is indicated or prescribed” (SAMHSA, 2016).

An opioid epidemic is also well documented in Canada, where a recent report on opioid prescription produced by the Canadian Centre on Substance Use and Addiction indicated that among the Canadian population who used opioid analgesics, 3% stated that they were using them for non-medical purposes [5].

The United Nations Office on Drugs and Crime (UNODC), in its recent world report on drugs, indicated that the use of drugs with no medical prescription is becoming a huge threat to public health and identified opioids as causing the most harm, representing 76% of deaths associated with psychoactive substance use. The report also drew attention to adolescence as a high-risk period for starting psychoactive substance consumption, which peaks in young adulthood (18–25 years) [6].

Prescription opioid misuse has become one of the most common ways drugs are consumed by young American adults and university students [7–11]. Research based on data from national health surveys, such as the NSDUH or Monitoring the Future (MTF), show annual American misuse prevalence estimates ranging from 4.8% to 7.5% among young adolescents, and from 7.6% to 13.2% among young adults [9]. The Centers for Disease Control and Prevention (CDC) recently analyzed data from the 2009–2019 Youth Risk Behavior Survey and found that in the year 2019, 7.2% of American adolescents admitted current prescription opioid misuse [10].

Several studies are questioning and analyzing whether Europe is currently in an opioid crisis similar to that of the US and Canada, based on data on the consumption and use of these prescription drugs [12–17]. Although consumption was high in the 1990s, the use of opioid analgesics has fallen in most European countries over the past 20 years. However, consumption levels vary among countries, with consumption of these drugs being ten times higher in Western and Northern Europe countries (Nordic states), exceeding 10,000 Defined Daily Doses (DDDs) per 1,000,000 inhabitants, compared to values declared by Southern and Eastern countries that are below 1,000 DDDs per 1,000,000 inhabitants (Rumania, Bulgaria and Albania) [18].

In Europe, opioid use contributes significantly to socio-sanitary costs associated with drug abuse, and the threat to public health from these drugs may indeed be increasing. The European Drug Report warns of rapidly increasing consumption of legal synthetic opioids—methadone, buprenorphine, and fentanyl. These particular opioids are the primary drugs for 22% of all patients treated for opioid use, and 19 European countries reported that more than 10% of opioid consumers who started a specialized treatment program were admitted due to problems mainly related to opioids other than heroin [19].

Among young Europeans, the average percentage non-medical use of prescription drugs is 9%, ranging from 2.8% to 23%, according to the latest European School Survey Project on Alcohol and Other Drugs (ESPAD). This non-medical use of prescribed drugs such as tranquilizers or sedatives and analgesics has become popular among adolescents, ranking as the second and third most abused substances, excluding tobacco and alcohol. That report states that painkillers are used to get high by about 4.0% of European students, with the highest prevalence in Slovakia (18%) and the Czech Republic (10%) [20].

Spain has much lower prescription opioid consumption rates compared with other Central Europe Countries, the United Kingdom and Canada. However, in the most recent decades, opioid use has increased significantly [17,18]. According to the Spanish Agency of Medicines and Health Products, consumption has risen from 10.02 DHD (defined daily dose/1,000 inhabitants per day) in 2010 to 19.94 DHD in 2020 [21].

However, we do not have specific data about the non-medical use of opioid prescription drugs among young Spanish adults, since there is not enough scientific evidence to address this issue. Therefore, the objective of our study was to use a representative national sample to describe the prevalence and factors associated with prescription opioid use and misuse among young adults living in Spain.

## Methods

### Sample and Procedure

We carried out a nationwide, cross-sectional epidemiological study on the use and misuse of prescription opioids among young Spanish adults aged 18–34 years. We used individualized secondary data provided by the Household Survey on Alcohol and Drugs in Spain (EDADES [Encuesta Domiciliaria sobre Alcohol y Drogas en España]) 2017–2018.

The EDADES survey has been conducted biannually by the Spanish National Drug Plan since 1995. It is a representative survey of the non-institutionalized population living in Spain aged 15–64 years designed to monitor alcohol and drug use including non-substance or behavioral addictions, along with perceptions and opinions about their use.

The EDADES survey includes a personal home interview, and information is gathered through a questionnaire. In the 2017–2018 version, for the first time, the survey included a question alluding to prescription opioid analgesic use and included the names of the active ingredients.

The questionnaire and methodology are similar to those used in the United States and other European Union countries. This allows for international comparisons.

Sampling is performed with three-stage non-replacement conglomerates and includes urban and rural populations from all Spanish autonomous communities and cities. EDADES Survey methodology specifics are available elsewhere [22].

Our study population comprised 6,382 young adults of both sexes aged 18–34 years residing in Spain when the survey took place.

### Measures

The dependent variable related to opioid analgesic use was based on responses to the following survey item: “Indicate which of the following Opioid Analgesics (drugs derived from morphine used to ease pain) you have used or consumed in the last twelve months”, referring to a list of drugs including Tramadol, Codeine, Morphine, Fentanyl, Oxycodone, Hydromorphone, Pethidine, Tapentadol, Methadone and Buprenorphine. Paracetamol, Ibuprofen, Aspirin, Nolotil, etc., were not included.

Opioid analgesic misuse was the dependent variable, with the dichotomous answer “yes” or “no,” for when consumption of these drugs during the previous twelve months took place without a physician’s prescription, but rather the drugs were obtained from a friend or family member, drug dealer or pusher, *via* the internet, etc.

Independent variables for the study were the main socio-demographic characteristics of the population, i.e., sex (male, female), age (18–24 years and 25–34 years), nationality (Spanish or immigrant), educational level (primary school, secondary school, higher education), occupational status (unemployed, employed or inactive), and monthly income (<€ 1,000; € 1,000–2,000; >€ 2,000). Concerning use of legal psychoactive substances, questions pertained to alcohol and tobacco use and to tranquilizer, sedative and sleeping pill misuse (dichotomous variable, yes/no). To gain information about the co-use of illegal psychoactive substances, questions were asked about use of cannabis, cocaine and other illicit psychoactive drugs (heroin, LSD, non-LSD, hallucinogenics, amphetamines) in the last 12 months (dichotomous variable, yes/no).

Risk perception regarding consumption of these drugs (two categories: No/few problems and quite a few/many problems), availability (two categories: Impossible/very difficult to obtain and Easy/very easy to obtain) and the self-perceived health (two categories: Very good/Good and Fair/Poor/Very poor) of respondents were also considered as study variables.

### Data Analysis

For data analysis purposes, we calculated the prevalence of prescription opioid use and misuse. Pearson’s χ2 test was used for bivariate comparison of proportions, and statistical significance was set at *p* < 0.05 (2-tailed) in all analyses.

To estimate the independent effect of each of the study variables on the consumption of prescription opioids, we obtained the corresponding adjusted odds ratio (AOR) and 95% Confidence Interval (CI) via multivariable logistic regression analysis. All variables that showed a significant association in the bivariate analysis were included in the multivariable analysis. Two models were generated: one to identify those factors associated with prescription opioid use in our sample and a second to identify the factors related to prescription opioid misuse in the young adult population.

Estimates were made using the svy function (survey command) of the Stata program (Stata Corp, College Station, Texas, USA. Stata/SE 16), which enabled us to incorporate the sample design and weights into all statistical calculations (descriptive, χ2 and logistic regression).

## Results

Descriptive characteristics of the 6,382 study subjects are shown in Table 1. During 2017 and 2018, the prevalence of prescription opioid use among young adults residing in Spain was 4.89%, with higher values among the female population (4.52% in men vs. 5.27% in women, *p* = 0.175). Table 2 contains prevalence data for prescription opioid use for both women and men, according to the variables included in the study, such as socio-demographic data, consumption of other legal and illegal psychoactive substances, data related to risk perception concerning drug availability and self-perceived health status.

**TABLE 1:** Sociodemographic characteristics of study population. Household Survey on Alcohol and Drugs (Spain. 2017–2018).

	N	(%)
Sex		
Male	3,241	50.78
Female	3,141	49.21
Age		
18–24 years	2,414	37.8
25–34 years	3,968	62.2
Nationality		
Immigrants	955	15.0
Spanish	5,406	85.0
Occupational status		
Employed	3,330	52.5
Unemployed	1,065	16.8
Inactive	1,952	30.8
Educational level		
Primary school	2,709	42.6
Secondary school	2,358	37.1
Higher education	1,292	20.3
Monthly income		
<1,000 €	881	21.4
1,000–2000€	2,108	51.3
>2,000€	1,124	27.3

**TABLE 2:** Prevalence of prescription opioids use, according to sociodemographic variables, use of licit and illicit psychoactive drugs and variables related with perceived health risk, perceived availability. Household Survey on Alcohol and Drugs (Spain. 2017–2018).

	Male	Female	Both sex	*p*-value^a^
	n (%)	n (%)	n (%)	
Age				
18–24 years	39 (3.15%)	52 (4.43%)	91 (3.77%)	0.087
25–34 years	107 (5.38%)	114 (5.76%)	221 (5.57%)	0.621
Nationality				
Immigrants	16 (3.63%)	20 (3.91%)	36 (3.79%)	0.838
Spanish	131 (4.68%)	145 (5.56%)	276 (5.1%)	0.146
Occupational status				
Employed	85 (4.82%)	94 (6.02%)	179 (5.38%)	0.138
Unemployed	32 (5.5%)	28 (5.71%)	60 (5.6%)	0.882
Inactive	29 (3.35%)	42 (3.87%)	71 (3.64%)	0.534
Educational level				
Primary school	66 (4.44%)	73 (5.94%)	139 (5.12%)	0.089
Secondary school	55 (4.58%)	52 (4.48%)	107 (4.53%)	0.901
Higher education	26 (4.7%)	40 (5.46%)	66 (5.13%)	0.541
Monthly income				
<1,000 €	18 (4.48%)	18 (3.77%)	36 (4.1%)	0.578
1,000–2,000€	62 (5.84%)	56 (5.38%)	118 (5.61%)	0.649
>2,000€	33 (5.63%)	43 (8.08%)	76 (6.79%)	0.118
Legal Psychoactive Substances				
Alcohol use in the past 12 months				
No	19 (3.28%)	32 (3.75%)	51 (3.56%)	0.635
Yes	128 (4.79%)	134 (5.83%)	261 (5.27%)	0.112
Tobacco use in the past 12 months				
No	56 (3.36%)	81 (4.11%)	137 (3.76%)	0.233
Yes	90 (5.78%)	85 (7.19%)	175 (6.39%)	0.147
Misuse of tranquilizers, sedatives, and sleeping pills				
No	135 (4.23%)	161 (5.2%)	297 (4.71%)	0.075
Yes	11 (27.74%)	4 (10.67%)	15 (19.36%)	0.040
Illegal Psychoactive Substances				
Cannabis use in the last 12 months				
No	95 (3.8%)	139 (4.92%)	234 (4.39%)	0.048
Yes	51 (6.97%)	26 (8.4%)	78 (7.4%)	0.458
Cocaine use in the last 12 months				
No	132 (4.23%)	162 (5.23%)	294 (4.73%)	0.067
Yes	15 (11.38%)	3 (7.81%)	18 (10.5%)	0.562
Other illicit psychoactive drug use in the last 12 months (heroin, LSD, non-LSD, hallucinogenic, amphetamine)				
No	130 (4.16%)	163 (5.25%)	292 (4.7%)	0.046
Yes	17 (13.63%)	3 (6.68%)	20 (11.92%)	0.314
Perceived health risk for opioids analgesic consumption				
No/few problems	44 (9.93%)	60 (15.78%)	104 (12.65%)	0.016
Quite a few/many problems	97 (4.05%)	96 (3.96%)	193 (4%)	0.877
Perceived availability of opioids analgesic consumption				
Impossible/very difficult to obtain	22 (1.93%)	47 (3.94%)	68 (2.96%)	0.007
Easy/very easy to obtain	114 (7.74%)	108 (8.2%)	222 (7.96%)	0.657
Self-assessment of health status				
Very good/Good	120 (3.93%)	141 (4.72%)	261 (4.32%)	0.134
Fair/Poor/Very poor	26 (14.57%)	25 (15.2%)	51 (14.87%)	0.871
Total consumption	147 (4.52%)	165 (5.27%)	312 (4.89%)	0.175

a Statistically significant differences on analyzing prevalence of opioids analgesic consumption, between young men and women (*p* < 0.05).

When we performed analyses based on independent variables, misuse of these drugs among young Spanish adults who stated they had used prescription opioids in the previous twelve months gave prevalence values of 13.4%, with different prevalence values among women and men (OR = 1.11; 95% CI: 0.55–2.22). Misuse prevalence values were also significantly higher among young adults who declared they had consumed cannabis and/or other illicit psychoactive drugs in the previous 12 months (Table 3).

**TABLE 3:** Prevalence of prescription opioids misuse, according to sociodemographic variables, use of licit and illicit psychoactive drugs and variables related with perceived health risk, perceived availability. Household Survey on Alcohol and Drugs (Spain. 2017–2018).

	n (%)	OR (95% CI)^a^
Sex		
Male	19 (12.79%)	1
Female	23 (13.95%)	1.11 (0.55–2.22)
Age		
18–24 years	28 (12.6%)	1
25–34 years	14 (15.35%)	1.25 (0.62–2.54)
Nationality		
Spanish	34 (12.55)	1
Immigrants	7 (20.27%)	1.78 (0.62–5.17)
Occupational status		
Employed	23 (12.77%)	1
Unemployed	11 (17.69%)	1.47 (0.62–3.52)
Inactive	8 (11.62%)	0.89 (0.39–2.04)
Educational level		
Primary school	18 (12.89%)	1
Secondary school	11 (10.7%)	0.80 (0.36–1.80)
Higher education	12 (18.71%)	1.55 (0.62–3.84)
Monthly income		
<1,000 €	4 (11.88%)	1
1,000–2,000€	15 (12.61%)	1.07 (0.39–2.94)
>2,000€	10 (13.25%)	1.13 (0.36–3.52)
Legal Psychoactive Substances		
Alcohol use in the last 12 months		
No	8 (15.74%)	1
Yes	34 (12.95%)	0.79 (0.34–1.85)
Tobacco use in the past 12 months		
No	19 (13.57%)	1
Yes	23 (13.28%)	0.98 (0.48–1.97)
Misuse of tranquilizers, sedatives, and sleeping pills in the last 12 months		
No	39 (13.01%)	1
Yes	3 (21.13%)	1.80 (0.50–6.38)
Illegal Psychoactive Substances		
Cannabis use in the last 12 months		
No	24 (10.15%)	1
Yes	18 (23.22%)	2.68 (1.28–5.59)
Cocaine use in the last 12 months		
No	37 (12.77%)	1
Yes	4 (23.51%)	2.09 (0.69–6.36)
Other illicit psychoactive drug use in the last 12 months (heroin, LSD, non-LSD, hallucinogenic, amphetamine)		
No	35 (11.96%)	1
Yes	7 (34.89%)	3.94 (1.38–11.24)
Perceived health risk for misuse of opioids analgesic		
No/few problems	22 (11.4%)	1
Quite a few/many problems	18 (17.51%)	1.65 (0.78–3.47)
Perceived availability of misuse of opioids analgesic		
Impossible/very difficult to obtain	25 (11.08%)	1
Easy/very easy to obtain	13 (18.96%)	1.87 (0.77–4.55)
Self-assessment of health status		
Very good/Good	34 (13.22%)	1
Fair/Poor/Very poor	7 (14.37%)	1.10 (0.45–2.68)
Total misuse	42 (13.4%)	NA

a Data are expressed as odds ratio (OR) and 95% confidence intervals (95% CI).

**FIGURE 1:**
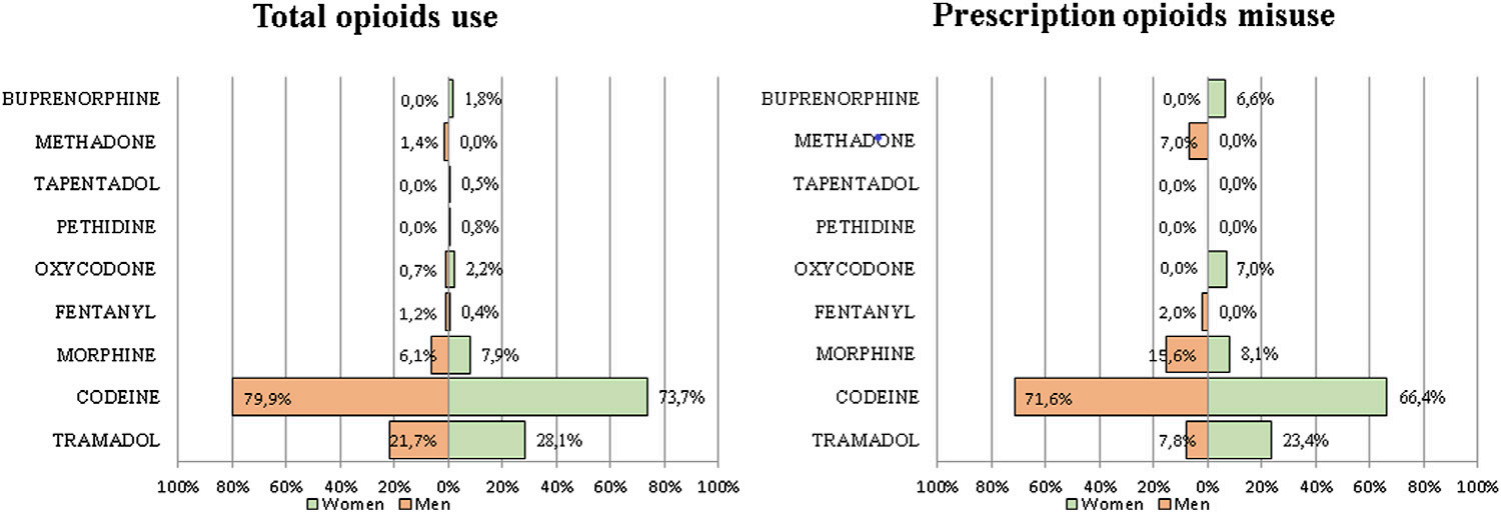
Prevalence of prescription opioids use and misuse by sex and generic drug. Household Survey on Alcohol and Drugs (Spain. 2017–2018).

For the first time, the EDADES survey included the names of the active ingredients of the prescription opioid analgesics about which the respondents were questioned. Figure 1 shows use and misuse for each active ingredient. Young adults of both sex made greater use of Tramadol, Morphine and Codeine, with Codeine the most used opioid (79.9% in men vs. 73.7% in women). When we analyzed misuse of prescription opioids, we found a sex difference. Although Codeine remained the most used drug for both sex, young women presented higher misuse values than men for Tramadol (23.4% in women vs. 7.8% in men), Oxycodone (7.0% in women vs. 0.0% in men) and Buprenorphine (6.0% in women vs. 0.0% in men). Methadone misuse appeared in 7% of young men.

Our multivariable analysis results for prescription opioid use, performed using logistic regression models (Table 4), showed that young women are more likely than men to use opioid analgesics (AOR = 1.32; 95% CI: 1.06–1.66).

**TABLE 4:** Multivariable logistic regression of prescription opioids use among young adults population in Spain. Household Survey on Alcohol and Drugs (Spain. 2017–2018).

	AOR^a^	95% CI
Sex		
Male	1	—
Female	1.32	(1.06–1.66)
Age		
18–24 years	1	—
25–34 years	1.06	(1.04–1.09)
Misuse of tranquilizers, sedatives, and sleeping pills in the last 12 months		
No	1	—
Yes	2.77	(1.53–5.01)
Cannabis use in the last 12 months		
No	1	-
Yes	1.47	(1.1–1.96)
Other illicit psychoactive drug use in the last 12 months (heroin LSD, non-LSD hallucinogenic, amphetamine)		
No	1	—
Yes	1.81	(1.07–3.09)
Perceived health risk for opioids analgesic use		
Quite a few/many problems	1	—
No/few problems	2.00	(1.57–2.56)
Perceived availability of opioids analgesic use		
Impossible/very difficult to obtain	1	—
Easy/very easy to obtain	2.74	(2.10–3.57)

a Data are expressed as adjusted odds ratio (AOR) and 95% confidence intervals (95% CI).

When we analyzed the variables associated with legal and illegal psychoactive substance use during the past 12 months, we found that tranquilizer, sedative and sleeping pill misuse (AOR = 2.77; 95% CI: 1.53–5.01), cannabis use (AOR = 1.47 95% CI: 1.1–1.96) and other illicit psychoactive drug use (AOR = 1.81; 95% CI: 1.07–3.09), were the variables independently and significantly associated with a greater probability of prescription opioid use.

The low risk perceived for using prescription opioids (AOR = 2.00; 95% CI: 1.57–2.56), as well as the ease with which they can be obtained (AOR = 2.74; 95% CI: 2.10–3.57), are usage predictors for these drugs.

The second multivariable logistic regression model created for prescription opioid misuse (Table 5) shows age variable as a protective factor for young adults aged between 25 and 34 years, with lower odds for misuse than those aged 18 to 24.

**TABLE 5:** Multivariable logistic regression of prescription opioids misuse among young adults population in Spain. Household Survey on Alcohol and Drugs (Spain. 2017–2018).

	AOR^a^	95% CI
Age		
18–24 years	1	—
25–34 years	0.94	(0.86–0.98)
Cannabis use in the last 12 months		
No	1	—
Yes	2.04	(1.03–4.05)
Other illicit psychoactive drug use in the last 12-month (heroin. LSD, non-LSD hallucinogenic. amphetamine)		
No	1	—
Yes	2.99	(1.10–8.15)

a Data are expressed as adjusted odds ratio (AOR) and 95% confidence intervals (95% CI).

Variables related to cannabis consumption and other illicit psychoactive drug use (AOR = 2.99; 95% CI: 1.10–8.15) in the last year showed a statistically significant association with a higher probability of prescription opioid analgesic misuse.

## Discussion

From a European perspective, this article is one of the first to describe and identify the factors associated with the use and misuse of prescription opioids among young adults residing in Spain. Opioid misuse is the consumption without a corresponding prescription, in a way other than as prescribed, or to achieve the experience and sensations they provide.

Although we have evidence that prescription opioid use has increased significantly in Spain over the last decade [21], when we compare our data with that from the US or Canada we observe that Spanish use is still significantly lower.

In our representative nationwide sample of young Spanish adults, 4.89% stated that they had used a prescription opioid in the past year, while 13.4% of them declared prescription opioid misuse. This prevalence of prescription opioid use among young Spanish adults aged 18 to 24 was below the 32% seen for such drug use among young adults aged 18 to 25 in the results of a recent study by Hudgins et al. carried out with data from the American NSDUH [23]. However, misuse prevalence values among young Americans (7.8%) are below the percentage declared by young Spanish adults in our study (12.6%). Similarly, data from the Canadian Centre on Substance Use and Addiction indicates that 8.4% of young adults aged 15–19 years, and 20% of those aged 20 to 24, had used an opioid analgesic drug in the last year [5].

Like Spain, other European countries, such as the Netherlands, the United Kingdom and the Nordic countries, have observed increases over the last decade prescription opioid use and misuse among the general population [24–26]. However, few studies have specifically focused on analyzing this consumption pattern among young European adults, which makes it very difficult to estimate the amount of actual misuse.

Some studies carried out in nearby countries, such as the one by Jeanne et al., the objective of which was to identify new substance use and abuse patterns among French adolescents, estimated the misuse of opioids such as methadone and buprenorphine at 0.6% [27]. Similarly, a study performed in Greece on a 15 to 19 year-old population found that 16.2% of these young adults had misused prescribed opioids and that 26% of those did so in order to feel good, get high, or try something new [28].

Codeine and Tramadol were the opioids most frequently consumed by young adults in our study, in line with the consumption data for these substances obtained in other studies carried out in both the USA and Europe [26,29–33]. There is a growing concern about the misuse of prescribed and over-the-counter (OTC) Codeine in our pharmacies, and this is becoming a significant public health issue. Wells et al., in a survey in three different countries with users of OTC drugs containing Codeine, found that 6% of Irish, 13% of South African and 16% of English respondents claimed that they had made weekly purchases of OTC drugs containing Codeine [30]. In Spain, according to the Spanish Agency of Medicines and Health Products, Tramadol is the most commonly used active ingredient among all opioid analgesics [21]. However, data from the USA, Canada and some European countries (Germany, Italy, Spain, and the UK) shows lower Tramadol non-medical use rates than those observed with conventional opioids. A recent study carried out in four European countries, with data from the Survey of Non-Medical Use of Prescription Drugs, indicated that in those countries, Tramadol misuse prevalence rates were lower than those observed for other opioids [31]. These results are consistent with those recently obtained by Reines et al. in a nationally representative sample of non-institutionalized Americans [32].

Gender differences related to prescription opioid use and misuse have evolved in recent years. Population surveys in the last decade have shown significant changes in the prevalence of opioid misuse between men and women [5,22,34]. Although misuse of these drugs has historically been more prevalent among men, gender differences in opioid analgesic misuse continue to decline, as shown by the results in the American study by McHugh et al., in which women, including young women, were more likely to misuse opioid analgesics than men (OR = 1.22; 95% CI: 1.09–1.35) [35]. Our results are consistent with that finding, since they show differentiated use and indicate that young women are more likely to use prescription opioids than men, and also present higher misuse values for Tramadol, Oxycodone, and Buprenorphine. However, female epidemiological opioid misuse in Spain is poorly characterized and the public health implications have not been sufficiently studied. The scientific evidence shows a need to look at several demographics as well as social variables to provide a more in-depth analysis of this situation among young women [36,37].

Sedative-hypnotic use is common among people who use opioids, but if this concomitant use takes place in recreational environments or non-medical situations, the risk of overdose and poisoning undoubtedly increases. When we analyzed use of these legal psychoactive substances among our study’s young population, we found that the odds ratio of tranquilizer, sedative and sleeping pill misuse during the last 12 months was 2.77 times greater among young adults who admitted to using prescription opioids (AOR = 2.77; 95% CI: 1.53–5.01). The results of a study by Tubbs et al. with data from the U.S. National Survey on Drug Use and Health for 2015–2018, indicated that people who had used an opioid in the last year were four times more likely to have consumed benzodiazepines (OR = 4.4; 95% CI: 3.61–5.4), with even higher associations found for opioid misuse [38]. Indeed, the results of an American study designed to reveal current trends regarding opioid drug poisoning among adolescents and young adults aged from 10 to 29 results indicated that benzodiazepines were the most commonly used drug, together with alcohol, in opioid poisonings [39].

It is well documented that the use of illegal psychoactive substances is a risk factor related to the onset of prescription drug misuse. Some studies, including ours, indicate that cannabis consumption is associated with a greater risk of prescription opioid misuse. Liang et al., in a sample of adults aged 18 and older, found that non-medical cannabis use was associated with a greater risk of prescription opioid misuse (OR = 3.15; 95% CI: 2.89–3.44) [40]. However, when the adolescent population of 12–17 year olds was looked at, as in the Carmona et al. study, results showed that 52.2% of young people who admitted to non-medical use of prescription opioids had also consumed cannabis, and this prevalence and potential danger were considered high because of the interactions between these two substances [41].

Our outcomes indicate that the use of illegal psychoactive substances other than cannabis is the strongest variable in the association with prescription opioid use and misuse (OR = 2.99; 95% CI: 1.10–8.15) among young Spanish adults. Grigsby et al., using NSDUH data, note that most respondents who stated they had misused prescription opioids also consumed other types of substances, including drugs such as cocaine, methamphetamine, etc. Their study also pointed out that 12 to 17-year-old adolescents and young adults aged 18 to 25 presented a higher risk of misusing these drugs as well as of consuming illicit drugs or of poly-drug use (*p* < 0.01) [3]. A study by Hudgins et al. carried out with an American adolescent and young adult population obtained similar results. Those who admitted to opioid use had a high use prevalence for cocaine (35.5%), hallucinogens (49.4%) and inhalants (30.4%) [23].

From these studies, we can deduce that prescription opioid misuse is common among adolescents and young adults and is frequently associated with the additional use of illegal psychoactive substances. This can be explained by taking account of the low risk perception of prescription opioid use and the relative ease of obtaining these substances in their environment expressed by the young adults in our study. A situation may also occur in which young adults who have previously been prescribed opioid analgesics have a lower risk perception of occasional misuse (OR = 0.61; 95% CI: 0.43–0.85, *p* = 0.01) for this type of drug, as reflected in a study by Romberg et al., which analyzed prescribed opioid misuse perceptions among young adults who might or might not have previously been prescribed opioids [42]. The factors favoring such perception and the subsequent increase in the rates of addictive behavior among young adults need to be identified.

The most common source for opioid misuse is often analgesics prescribed to relatives and friends. Opioids are prevalent in homes in our communities, and relatives who are insufficiently aware of the risks of misuse may be enabling their use by other members of the household, as an American study by Garbutt et al. showed. That study pointed out that 30% of parents in the study did not know that their children were misusing prescription opioids [43]. In addition, we cannot rule out the existence of a black market for these drugs in our country and within certain juvenile contexts. Even though the Spanish Health System has implemented control mechanisms in recent years aimed at preventing inappropriate opioid prescription in order to limit opioid abuse, we cannot turn a blind eye to this incipient reality among young Spanish adults. Prevention efforts must take into account that the young people in our study who use prescription opioids report that they do not find it difficult to obtain these drugs.

One strength of our study is that this article is one of the first to describe and identify the factors associated with prescription opioid use and misuse among young Spanish adults, using a nationwide representative sample. This EDADES survey, for the first time in its series, has incorporated the generic names of prescription opioid analgesics, which has allowed us to identify the drugs involved in the misuse.

The present study has certain limitations. The first relates to the use of surveys on drug consumption associated with the cross-sectional nature of the study data, since it does not allow establishment of the direction of the associations found. Second, the use of self-reported data means that the prevalence results obtained for prescription opioid misuse patterns may be under-reported by the young survey takers, due to socio-cultural connotations surrounding drug use. Finally, the small number of cases, especially of opioid misuse, is clearly a limitation of the study and may partly explain the lack of significance of some independent variables.

It should be noted that the structure of the Spanish National Health System, with its universal coverage, has a series of characteristics that hinder misuse of these drugs, such as the prohibition on advertising opioid drugs and the use of electronic prescriptions that prevents opioids being accumulated.

### Conclusion

In Spain, prescription opioid use among young adults was found to be 4.89%. The misuse of these drugs among young Spaniards who admitted to using prescription opioids during the last year was at 13.4%, with higher percentages for young women. Codeine and Tramadol are the most frequently consumed prescription opioids. The misuse of tranquilizers, sedatives, and sleeping pills, along with the use of cannabis and other illicit psychoactive drugs are correlates of prescription opioid use. Factors associated with prescription opioid misuse were the use of cannabis and illicit psychoactive drugs in the previous year.

Our findings have important implications for public health, since they offer scientific evidence concerning prescription opioid misuse among young Spanish adults. Up to now, there has not been sufficient scientific evidence in Spain to address the subject adequately.

Even though we cannot draw parallels between the so-called “opioid epidemic” affecting countries such as the United States or Canada, we can see that we may be on the threshold of similar problems to those faced by young adults in those countries. Understanding the reasons for prescription opioid misuse is critical, as this will facilitate decision-making for greater control in the prescription of opioids by health professionals and in the possibility of obtaining them through illicit sources.
